# *Notes from the Field:* Emergency Department Visits for Unsupervised Pediatric Melatonin Ingestion — United States, 2019–2022

**DOI:** 10.15585/mmwr.mm7309a5

**Published:** 2024-03-07

**Authors:** Devin I. Freeman, Jennifer N. Lind, Nina J. Weidle, Andrew I. Geller, Nimalie D. Stone, Maribeth C. Lovegrove

**Affiliations:** ^1^Oak Ridge Institute for Science and Education, Oak Ridge, Tennessee; ^2^Division of Healthcare Quality Promotion, National Center for Emerging and Zoonotic Infectious Diseases, CDC; ^3^Chenega Enterprise Systems & Solutions, Atlanta, Georgia.

**Summary**

**What is already known about this topic?**

Unsupervised exposures of infants and young children to melatonin have increased substantially in recent years.

**What is added by this report?**

During 2019–2022, melatonin was implicated in approximately 11,000 (7%) emergency department visits among infants and young children for unsupervised medication ingestions. Many incidents involved ingestion of flavored products (e.g., gummy formulations).

**What are the implications for public health practice?**

Approximately 11,000 emergency department visits for unsupervised melatonin ingestions by infants and young children during 2019–2022 highlights the importance of educating parents and other caregivers about keeping all medications and supplements (including gummies) out of children’s reach and sight.

The prevalence of melatonin use by U.S. adults quintupled from 0.4% during 1999–2000 to 2.1% during 2017–2018 (*1*). This rise coincided with a 530% increase in poison center calls for pediatric melatonin exposures during 2012–2021 and a 420% increase in emergency department (ED) visits for unsupervised melatonin ingestion by infants and young children during 2009–2020 (*2*,*3*). CDC analyzed public health surveillance data to describe circumstances involved in these ingestions to help guide development of interventions.

## Investigations and Outcomes

Data from the National Electronic Injury Surveillance System – Cooperative Adverse Drug Event Surveillance Project were used to identify cases of ED visits for unsupervised melatonin ingestion by infants and children aged ≤5 years during 2019–2022, based on the treating clinician’s diagnosis and supporting documentation in the ED record.* Case narratives were used to code circumstances and details about ingested melatonin products. Cases were weighted to allow calculation of national estimates and corresponding 95% CIs. SAS software (version 9.4; SAS Institute) SURVEYMEANS was used to account for sample weights and complex sample design. This activity was reviewed by CDC, deemed not research, and was conducted consistent with applicable federal law and CDC policy.^†^

Based on 295 cases, an estimated 10,930 ED visits (95% CI = 7,609–14,251) occurred for unsupervised melatonin ingestion by infants and children aged ≤5 years in the United States during 2019–2022 (Table), accounting for 7.1% of all ED visits for unsupervised medication exposures by persons in this age group. Approximately one half (52.4%) of all estimated ED visits for melatonin ingestion by infants and children aged ≤5 years involved children aged 3–5 years, and most (93.5%) did not result in hospitalization. Melatonin was the only medication involved in 90.2% of ED visits for melatonin ingestions.

**TABLE:** Cases and national estimates of emergency department visits for unsupervised melatonin ingestion by infants and children aged ≤5 years — United States, 2019–2022

Characteristic	No. of cases	National estimates of emergency department visits	
		No.	% (95% CI)
**Total**	**295**	**10,930**	**100**
**Year**			
2019	42	2,032	18.6 (10.7–26.4)
2020	74	3,294	30.1 (19.8–40.5)
2021	78	2,135	19.5 (11.7–27.3)
2022	101	3,469	31.7 (18.3–45.2)
**Age group, yrs**			
0–2	159	5,201	47.6 (38.0–57.2)
3–5	136	5,729	52.4 (42.8–62.0)
**Sex**			
Female	123	4,569	41.8 (33.2–50.4)
Male	172	6,360	58.2 (49.6–66.8)
**Race**			
Black or African American	82	1,946*	17.8 (7.6–28.0)
White	117	5,718	52.3 (38.7–65.9)
Other or not specified	96	3,266	29.9 (16.6–43.2)
**Hospitalized**			
Yes	19	—^†^	—^†^
No	276	10,223	93.5 (89.3–97.8)
**Additional implicated medications**			
No	269	9,854	90.2 (84.8–95.5)
**Route^§^**			
Oral ingestion	291	10,782	98.6 (96.3–100.0)
**Dosage form^¶^**			
Solid	278	10,465	95.7 (92.3–99.2)
Gummy	140	4,953	47.3 (35.5–59.2)
Chewable tablet	19	—^†^	—^†^
Unspecified solid dosage form	119	5,146	49.2 (37.1–61.3)
No. of units accessed**			
1–9	81	3,211	30.7 (21.9–39.5)
10–19	35	1,423	13.6 (8.3–18.9)
≥20	59	2,320	22.2 (14.1–30.2)
Unspecified	103	3,510	33.5 (23.2–43.9)
**Intended age group of formulation^††^**			
Family or adult	128	5,210	47.7 (39.6–55.8)
Pediatric	21	—^†^	—^†^
Unspecified	146	4,919	45.0 (36.7–53.3)
**Container type**			
Bottle	94	3,590	32.8 (23.1–42.6)
Other or no container	28	—^†^	—^†^
Unspecified	173	6,188	56.6 (46.0–67.2)

**Source:** National Electronic Injury Surveillance System – Cooperative Adverse Drug Event Surveillance Project, CDC.

* Coefficient of variation = 32.9%. Estimates with a coefficient of variation >30% might be statistically unstable.

^†^ Estimates based on <20 cases and total estimates of <1,200 emergency department visits are considered statistically unreliable and are not shown.

^§^ Four cases (not shown) were for removal of a pill or tablet from the patient’s nose.

^¶^ Three cases (not shown) involved ingestion of liquid melatonin, and 14 involved ingestion of a melatonin product with an unspecified dosage form.

** Only assessed for emergency department visits involving ingestion of solid dosage form melatonin products.

^††^ Based on information from case narratives as well as information about available products. For example, the age group of formulation was coded as “pediatric” for cases specifying a specific product that is intended for pediatric use. Age group of formulation was coded as “family or adult” for cases specifying a product intended for family or adult use, a specific adult recipient, or a dosage strength >1 mg per unit.

A solid dosage form product was accessed by infants and children aged ≤5 years in 95.7% of ED visits for melatonin ingestions by persons in this age group. Gummy formulations (47.3%) were the most commonly documented dosage form; however, an unspecified solid formulation was documented in approximately one half (49.2%) of visits. Access to ≥10 units (e.g., gummies or tablets) was documented in more than one third (35.8%; 95% CI = 28.6%–43.0%) of visits for solid melatonin ingestions. Ingestion of adult or family formulations^§^ of melatonin was documented in 47.7% of visits; however, intended age group of formulation was not specified in 45.0% of visits. At least 32.8% of infants and children accessed melatonin from a bottle; however, container type was not documented for 56.6% of visits.

## Preliminary Conclusions and Actions

During 2019–2022, melatonin was implicated in 7% of all ED visits for unsupervised medication exposures by infants and young children. Few visits were found to result in hospitalization in this study. Similarly, a recent study of poison center calls found that 98% of pediatric melatonin exposures resulted in minimal or no effects and increases in hospitalizations for pediatric melatonin ingestion coincided with increased use (*2*). However, another recent investigation of melatonin products found that the actual content of the melatonin product was not always the same as the labeled ingredients or strength, and these discrepancies in ingredients or strength could pose additional risk.^¶^

Approximately one half of visits for melatonin ingestions by infants and children aged ≤5 years involved children aged 3–5 years, whereas most visits for unsupervised medication exposures overall involve infants and children aged 1–2 years (*3*). At least half of ED visits for melatonin ingestions involved flavored products (gummies or chewable tablets) that are frequently used by (*4*) and might appeal to young children.

Melatonin products do not require child-resistant packaging,** although such packaging can be voluntarily implemented. Among ED visits with documentation of container type, approximately three quarters involved melatonin accessed from bottles, suggesting that infants and children opened bottles or that bottles were not properly closed. Selecting products with child-resistant packaging might be advisable in homes with young children.

Surveillance data have limitations. Analyzing only cases resulting in ED visits likely underestimates overall melatonin ingestions by infants and young children. Detailed narrative information was not always documented; therefore, misclassification might occur, and involvement of specific product types or circumstances might be higher than reported.

The occurrence of approximately 11,000 ED visits for unsupervised melatonin ingestions by infants and young children during 2019–2022 highlights the continued need to educate parents and other caregivers about the importance of keeping all medications and supplements (including gummies) out of children’s reach and sight (*5*). The Up and Away Campaign^††^ is an initiative led by CDC in collaboration with other government and nongovernmental partners to educate families about the importance of safe medicine storage around young children.

## Footnotes

*
https://health.gov/healthypeople/objectives-and-data/data-sources-and-methods/data-sources/national-electronic-injury-surveillance-system-cooperative-adverse-drug-event-surveillance-project-neiss-cades

† 45 C.F.R. part 46.102(l)(2), 21 C.F.R. part 56; 42 U.S.C. Sect. 241(d); 5 U.S.C. Sect. 552a; 44 U.S.C. Sect. 3501 et seq.

§ Products that are family formulations include dosing instructions for both adults and children aged <12 years and are not marketed specifically for pediatric use (e.g., the product name does not indicate “children’s”).

¶
https://doi.org/10.1001/jama.2023.2296

** 16 CFR Sect. 1700.14; 38 FR 21247, amended in 41 FR 22266; and 48 FR 57480.

††
https://www.upandaway.org
